# Multidimensional Determinants of Sexual Dysfunction in Multiple Sclerosis: Incremental Insights from a Hierarchical Model

**DOI:** 10.3390/jcm15062304

**Published:** 2026-03-18

**Authors:** Yusuf Sarı, Neslihan Cansel, Mehmet Tecellioğlu

**Affiliations:** 1 Department of Psychiatry, İnönü University, Turgut Özal Medical Center, Malatya 44100, Türkiye; yusuf.sari1@saglik.gov.tr; 2 Department of Neurology, İnönü University, Turgut Özal Medical Center, Malatya 44100, Türkiye; mehmet.tecellioglu@inonu.edu.tr

**Keywords:** multiple sclerosis, sexual dysfunction, fatigue, hierarchical regression, MSISQ-19, quality of life

## Abstract

**Background/Objectives**: Sexual dysfunction (SD) is a common yet underrecognized consequence of multiple sclerosis (MS) with multidimensional determinants spanning neurological, physical, and psychosocial domains. However, the relative and incremental contribution of fatigue compared with disability and psychological factors remains insufficiently clarified. This study aimed to evaluate the prevalence and severity of sexual dysfunction in MS and to identify independent and incremental predictors using a hierarchical analytical framework. **Methods**: In this cross-sectional case–control study, 140 patients with MS and 140 age- and sex-matched healthy controls were assessed. Sexual functioning was evaluated using the Arizona Sexual Experience Scale (ASEX) and the Multiple Sclerosis Intimacy and Sexuality Questionnaire-19 (MSISQ-19). Fatigue severity, anxiety, depression, body image, self-esteem, disability (EDSS), and quality of life were measured using validated instruments. Hierarchical multiple linear regression analyses were conducted within the MS cohort to determine independent predictors of MSISQ-19 domain and total scores and to evaluate the incremental contribution of fatigue. **Results**: Sexual dysfunction was significantly more prevalent in the MS group compared with controls (70.7% vs. 13.6%, *p* < 0.001). Patients with MS demonstrated higher fatigue, anxiety, depression, and poorer body image, self-esteem, and quality of life (all *p* < 0.001). Within the MS cohort, individuals with sexual dysfunction exhibited greater symptom burden and disability. Hierarchical regression analyses showed that fatigue produced the largest increase in explained variance across all MSISQ-19 domains (ΔR^2^ range: 0.11–0.19) and remained the strongest independent predictor (β range: 0.29–0.42; *p* ≤ 0.001). Anxiety retained independent associations with selected domains, whereas disability indices did not remain independently significant after adjustment. **Conclusions**: Sexual dysfunction in MS reflects a multidimensional burden extending beyond neurological impairment. Fatigue demonstrated the most consistent associations across sexual dysfunction domains, with anxiety contributing to selected aspects of functioning. Given the cross-sectional design, these findings should be interpreted as associations rather than causal relationships. Multidimensional assessment incorporating fatigue and psychological factors may improve the identification of clinically relevant and potentially modifiable determinants within comprehensive MS care.

## 1. Introduction

Multiple sclerosis (MS) is a chronic, immune-mediated inflammatory and neurodegenerative disorder of the central nervous system that predominantly affects young and middle-aged adults [1,2,3]. The disease course is characterized by demyelination, axonal loss, and progressive neurological impairment, resulting in variable degrees of physical disability, cognitive dysfunction, and psychosocial burden. Although clinical monitoring traditionally emphasizes relapse activity and motor disability, accumulating evidence suggests that many of the most impactful consequences of MS arise from symptom domains not fully captured by conventional neurological metrics such as the Expanded Disability Status Scale (EDSS) [1,4,5].

Sexual dysfunction (SD) is among the most prevalent yet underrecognized complications of MS, with reported rates ranging from 40% to 80%. Conceptually, SD in MS has been framed within a tripartite model encompassing primary mechanisms (direct neurogenic disruption of spinal and cortical pathways), secondary mechanisms (MS-related physical symptoms such as fatigue, spasticity, and mobility limitations), and tertiary mechanisms (psychosocial consequences including altered body image, diminished self-esteem, anxiety, and depression) [6,7]. While this framework provides a clinically meaningful taxonomy, empirical studies have frequently evaluated these components separately, limiting understanding of their relative contributions within an integrated explanatory model [8,9]. Importantly, sexual dysfunction in MS affects both men and women, although the clinical manifestations may differ by sex. Men with MS may experience erectile dysfunction, ejaculatory difficulties, and reduced libido, whereas women more commonly report decreased arousal, lubrication difficulties, orgasmic dysfunction, and pain during intercourse. Despite these differences, many studies have examined sexual dysfunction as a single construct without fully exploring potential sex-related variations in determinants and symptom expression.

Fatigue represents one of the most pervasive and disabling symptoms in MS, affecting up to 80% of patients. Importantly, fatigue severity often demonstrates only modest correlation with structural neurological disability, suggesting partially independent pathophysiological pathways involving inflammatory activity, central network inefficiency, autonomic dysregulation, and reward-processing mechanisms [10,11]. In addition to reflecting reduced physical energy, fatigue may interfere with sexual initiation, reduce perceived sexual competence, and alter autonomic and motivational processes that support sexual drive and arousal. Despite this theoretical plausibility, fatigue has rarely been examined in terms of its incremental explanatory value for disease-specific sexual dysfunction after simultaneous adjustment for neurological disability and psychological distress [12,13].

Psychological variables, including anxiety, depressive symptoms, body image dissatisfaction, and reduced self-esteem, are also highly prevalent in MS and have been individually associated with impaired sexual functioning. However, much of the existing literature relies on bivariate associations or generic sexual function measures, limiting insight into how these constructs interact within the broader clinical context [14,15]. Only a limited number of studies have applied hierarchical multivariable approaches to determine whether psychosocial factors exert independent effects beyond neurological impairment and whether fatigue contributes additional explanatory power across disease-specific sexual dysfunction domains [14,16].

Consequently, an important knowledge gap persists; it remains unclear whether sexual dysfunction in MS is primarily driven by neurological disability, psychological distress, fatigue as a partially independent symptom cluster, or the interaction of these factors within a multidimensional framework. Clarifying the relative and incremental contribution of these determinants is essential for identifying clinically actionable targets that extend beyond traditional neurological management [17,18].

Although comparisons with healthy individuals may help contextualize the prevalence and severity of sexual dysfunction, the primary analytical focus of the present study is to identify determinants of disease-specific sexual dysfunction within the MS population.

The present study addresses this gap by simultaneously evaluating neurological disability, fatigue severity, anxiety, depression, body image perception, self-esteem, and quality of life within a structured hierarchical analytical framework. Using the disease-specific Multiple Sclerosis Intimacy and Sexuality Questionnaire-19 (MSISQ-19), we aimed (1) to compare the prevalence and severity of sexual dysfunction between patients with MS and age- and sex-matched healthy controls, and (2) to identify independent and incremental predictors of primary, secondary, tertiary, and total MSISQ-19 scores within the MS population. In this framework, fatigue was entered as a symptom-level construct prior to psychosocial variables in order to examine its independent contribution to sexual dysfunction beyond neurological disability and before broader emotional factors were considered. We hypothesized that fatigue severity would demonstrate consistent domain-wide associations and explain meaningful additional variance beyond neurological disability and psychosocial variables.

## 2. Materials and Methods

### 2.1. Study Design and Participants

This cross-sectional case–control study was conducted as a collaborative investigation between the Departments of Neurology and Psychiatry at Turgut Özal Medical Center, İnönü University, between March and October 2021.The study aimed to evaluate sexual dysfunction and its associated clinical and psychological correlates in patients with multiple sclerosis (MS) compared with age- and sex-matched healthy controls. The control group was included primarily to provide a reference comparison for the prevalence and severity of sexual dysfunction and related psychological measures, whereas the primary analytical models were conducted within the MS cohort to identify determinants of disease-specific sexual dysfunction.

A total of 140 patients with a confirmed diagnosis of MS who were under regular follow-up at the neurology outpatient clinic were consecutively recruited. The control group consisted of 140 healthy individuals matched for age and sex, with no known neurological, psychiatric, or chronic systemic disorders.

Eligible patients were between 18 and 49 years of age, literate, and reported an active sexual life. All patients met the 2017 revised McDonald criteria for MS for at least six months prior to enrollment and had not experienced a clinical relapse within the preceding month. Individuals with clinically significant cognitive impairment that could interfere with questionnaire completion were excluded. Additional exclusion criteria included pregnancy, breastfeeding, or menopause in female participants; use of medications known to affect sexual functioning; diabetes mellitus; cardiovascular disease; other neurological disorders; severe psychiatric conditions; and illiteracy. Healthy controls met the same age and literacy criteria and were screened to exclude any neurological, psychiatric, or chronic medical illness.

Ethical approval was obtained from the Scientific Research and Publication Ethics Committee of İnönü University (Decision No: 2021/1519). The study was conducted in accordance with the Declaration of Helsinki. Written informed consent was obtained from all participants, and no financial compensation was provided.

### 2.2. Data Collection Procedure

All participants were evaluated in a private and confidential setting at the Multiple Sclerosis and Psychiatry outpatient clinics. Each participant underwent a face-to-face assessment conducted by a neurologist and subsequently by a psychiatrist to ensure comprehensive clinical evaluation.

Demographic and clinical data were recorded using a standardized data collection form. Clinical characteristics of patients with MS, including disease duration, relapse number, and treatment history, were obtained from medical records and verified during interviews. Neurological disability was assessed using the Expanded Disability Status Scale (EDSS) by an experienced neurologist trained in its application.

All self-report questionnaires were completed directly by participants in a private setting. When necessary, trained research staff provided clarification regarding questionnaire instructions without influencing responses. This procedure ensured standardized administration while preserving the self-report nature of the instruments.

### 2.3. Measures

A structured sociodemographic and clinical information form was developed to collect age, sex, education level, marital status, employment status, income level, smoking status, disease duration, relapse number, and treatment characteristics.

Sexual functioning was evaluated using the Arizona Sexual Experience Scale (ASEX) [19] and the Multiple Sclerosis Intimacy and Sexuality Questionnaire-19 (MSISQ-19) [20]. The ASEX is a five-item Likert-type instrument assessing sexual drive, arousal, erection/lubrication, ability to reach orgasm, and satisfaction from orgasm. A cutoff score ≥ 11 was used to indicate sexual dysfunction. The MSISQ-19 is a disease-specific measure assessing sexual dysfunction in MS across primary (neurogenic), secondary (symptom-related), and tertiary (psychosocial) domains.

Health-related quality of life was assessed using the Multiple Sclerosis Quality of Life-54 (MSQOL-54) [21], which provides physical and mental composite scores, with higher scores indicating better quality of life.

Fatigue severity was measured using the Fatigue Severity Scale (FSS) [22]. The mean score represents overall fatigue severity, and a score ≥ 4 indicates clinically significant fatigue.

Body image perception was evaluated using the Body Image Scale, with higher scores indicating greater body dissatisfaction. Self-esteem was assessed using the Rosenberg Self-Esteem Scale short form [23], with higher scores reflecting higher self-esteem.

Depressive and anxiety symptoms were evaluated using the Beck Depression Inventory (BDI) and Beck Anxiety Inventory (BAI), respectively [24].

### 2.4. Statistical Analysis

Statistical analyses were performed using IBM SPSS Statistics version 26.0. Normality was assessed using the Shapiro–Wilk test and graphical inspection. Continuous variables were presented as mean ± standard deviation or median (min–max), and categorical variables as frequencies and percentages.

Between-group comparisons (MS vs. controls) were conducted using independent samples *t*-tests or Mann–Whitney U tests, as appropriate. Within-group comparisons among patients with MS (sexual dysfunction present vs. absent) were performed using the same approach. Categorical variables were compared using chi-square or Fisher’s exact tests, and odds ratios with 95% confidence intervals were calculated for categorical outcomes.

Hierarchical multiple linear regression analyses were performed within the MS cohort to identify independent predictors of MSISQ-19 primary, secondary, tertiary, and total scores. Variables were entered in theoretically informed blocks: (1) demographic factors, (2) clinical disease characteristics including EDSS, (3) fatigue severity, and (4) psychosocial variables (anxiety, depression, body image, and self-esteem). Standardized beta coefficients (β), R^2^, and change in R^2^ (ΔR^2^) were reported to evaluate incremental explanatory power.

Regression assumptions were examined, including multicollinearity (variance inflation factor < 3), homoscedasticity, residual normality, and independence of errors (Durbin–Watson statistic). All tests were two-tailed, and *p* < 0.05 was considered statistically significant. Given the hypothesis-driven nature of the analyses, no formal correction for multiple comparisons was applied.

Fatigue severity was entered before psychosocial variables based on its conceptual role as a core symptom of MS that may influence motivational processes, physiological arousal, and behavioral engagement prior to broader emotional responses. This modeling strategy allowed the evaluation of fatigue as a symptom-level determinant independent of both neurological disability and subsequent psychological constructs.

## 3. Results

A total of 140 patients with MS and 140 age- and sex-matched healthy controls were included in the analysis. The two groups were comparable with respect to age, sex distribution, and educational level (all *p* > 0.05).

Within the MS cohort, the median disease duration was 7 years (0.5–15), the median relapse number was 3 (1–20), and the median EDSS score was 2.0 (1.0–6.5), indicating predominantly mild-to-moderate neurological disability. Sociodemographic and disease-related characteristics of the study population are presented in Table 1.

Sexual dysfunction was significantly more prevalent among patients with MS compared with healthy controls (70.7% vs. 13.6%, *p* < 0.001), corresponding to a markedly elevated odds ratio (OR: 15.3; 95% CI: 8.5–27.4). Median ASEX scores were also significantly higher in the MS group compared with controls (15 [5–28] vs. 5 [5–21], *p* < 0.001), indicating greater impairment in sexual functioning.

Patients with MS exhibited significantly higher anxiety scores (BAI: 10 [2–35] vs. 4 [0–18], *p* < 0.001) and depression scores (BDI: 16 [3–32] vs. 4 [0–19], *p* < 0.001) compared with controls. Fatigue severity was also significantly greater in the MS group (FSS: 4.9 [1.2–7.7] vs. 1.6 [0.9–4.4], *p* < 0.001). Clinically significant fatigue (FSS ≥ 4) was markedly more common among patients with MS (66.4% vs. 5.0%, OR: 37.6; 95% CI: 15.8–89.4; *p* < 0.001).

In addition, MS patients demonstrated significantly poorer body image perception (108 [48–182] vs. 184 [76–200], *p* < 0.001) and lower self-esteem scores (30 [12–40] vs. 35 [24–40], *p* < 0.001). Both physical and mental components of health-related quality of life were also significantly reduced in the MS group (MSQOL-54 Physical: 58.9 [22.8–92.2] vs. 96.9 [46.1–99.5], *p* < 0.001; MSQOL-54 Mental: 56.3 [17.7–90.1] vs. 93.0 [29.2–97.6], *p* < 0.001). These comparisons between the MS and control groups are summarized in Table 2.

Within the MS cohort, patients classified as having sexual dysfunction differed significantly from those without sexual dysfunction across multiple domains (Table 3). Patients with sexual dysfunction were older (38.3 ± 7.9 vs. 35.0 ± 6.4 years, *p* = 0.010) and exhibited higher neurological disability scores (EDSS: *p* = 0.002). They also demonstrated significantly higher anxiety (*p* < 0.001), depression (*p* < 0.001), and fatigue severity scores (*p* < 0.001). Furthermore, individuals with sexual dysfunction reported poorer body image perception (*p* < 0.001), lower self-esteem (*p* < 0.001), and significantly reduced physical and mental quality-of-life scores (both *p* < 0.001).

Hierarchical multiple linear regression analyses were performed to identify independent predictors of MSISQ-19 primary, secondary, tertiary, and total scores within the MS cohort. Across all models, the inclusion of fatigue severity (FSS) resulted in the largest increase in explained variance (ΔR^2^ range: 0.11–0.19). In the fully adjusted models, fatigue remained the strongest independent predictor across all MSISQ-19 domains (β range: 0.29–0.42; all *p* ≤ 0.001). Anxiety demonstrated an additional independent association with MSISQ-19 primary and total scores, whereas other psychosocial variables did not retain independent significance after adjustment.

Final model R^2^ values ranged from 0.334 to 0.453, indicating moderate explanatory power. Regression diagnostics indicated no evidence of problematic multicollinearity (all VIF < 3), and residual independence was acceptable (Durbin–Watson range: 1.70–2.27). The hierarchical regression results are summarized in Table 4.

Correlation analyses further supported the multidimensional structure of sexual dysfunction in MS. MSISQ-19 subscales showed moderate positive correlations with anxiety and depressive symptoms and negative correlations with self-esteem and health-related quality-of-life indices. Fatigue and neurological disability demonstrated distinct association patterns across MSISQ-19 domains. Spearman correlation coefficients are presented in Table 5.

To enhance transparency of domain-specific findings, detailed MSISQ-19 descriptive statistics and fully adjusted hierarchical regression coefficients are provided in Supplementary Tables S1 and S2. These supplementary analyses support the primary results by demonstrating the relative prominence of the secondary domain and the attenuation of demographic and disability effects after the inclusion of fatigue and psychosocial variables, while fatigue consistently retained the strongest independent association across models.

## 4. Discussion

The present study demonstrates that sexual dysfunction in multiple sclerosis (MS) should not be interpreted merely as a secondary complaint but rather as a multidimensional clinical phenomenon shaped by neurological, psychological, and functional factors. Compared with age- and sex-matched healthy controls, individuals with MS exhibited substantially higher prevalence and severity of sexual dysfunction alongside greater fatigue, anxiety, depressive symptoms, body image disturbance, reduced self-esteem, and poorer health-related quality of life.

Although specific sexual complaints were not analyzed individually, domain-level MSISQ-19 descriptive data (Supplementary Table S1) indicated relatively greater involvement of the secondary domain. This finding suggests that symptom-related mechanisms—particularly fatigue and functional burden—may represent a dominant pathway shaping sexual functioning in this cohort. The observed pattern partially contrasts with studies emphasizing tertiary mechanisms and may reflect the relatively mild-to-moderate disability distribution, sociocultural reporting variability, and the domain-wide prominence of fatigue demonstrated in hierarchical analyses [25,26].

Age and disability were associated with sexual dysfunction in unadjusted comparisons; however, their effects attenuated in hierarchical models after fatigue and psychological variables were introduced. This finding suggests that demographic and global disability indices may function as proxies for broader symptom burden rather than direct determinants of sexual functioning. EDSS, while clinically essential, may not fully capture autonomic dysfunction, sensory alterations, medication effects, relational context, or fatigue-related limitations that more directly influence intimate functioning [17,27]. Furthermore, the relatively young age distribution and the predominance of mild-to-moderate disability in our cohort may have reduced the independent contribution of disability-related variables in multivariable analyses.

The most consistent finding of the present study was the domain-wide prominence of fatigue. Fatigue produced the largest incremental increase in explained variance and remained the strongest independent predictor across all MSISQ-19 domains. However, given the cross-sectional design of the study, fatigue should not be interpreted as a causal determinant of sexual dysfunction. Rather, fatigue may represent a central component of overall symptom burden that interacts with both neurological impairment and psychological distress. These results support conceptualizations of fatigue as a central symptom extending beyond energy depletion to influence motivation, perceived bodily competence, physiological arousal, and relational engagement. Mechanistically, fatigue may reduce sexual initiation, shorten interaction duration, increase avoidance behaviors, and diminish emotional availability. Consequently, interventions targeting fatigue—behavioral, psychological, or pharmacological—may yield secondary benefits for sexual functioning [28,29,30].

Among psychological variables, anxiety retained independent associations with selected domains, highlighting its potential role in performance concerns, avoidance patterns, and heightened interpretation of bodily sensations as threatening. In contrast, depressive symptoms, although elevated at the group level, did not remain independently associated after adjustment, likely reflecting shared variance with fatigue and anxiety [31,32].

Lower body image and self-esteem among individuals with sexual dysfunction further underscore the clinical relevance of tertiary mechanisms. However, their attenuated effects in final models suggest that these constructs may operate through broader symptom burden and emotional distress rather than exerting fully independent effects. Physical changes related to MS—including mobility limitations, visible assistive devices, urinary concerns, and weight fluctuations—may influence perceived attractiveness and relational confidence, shaping sexual experiences even when statistical independence is not observed [33,34].

Quality-of-life findings reinforce the integrative nature of sexual health in MS. Marked reductions in both physical and mental MSQOL-54 components, particularly among individuals with sexual dysfunction, suggest that sexual functioning is both a consequence and potential contributor to overall well-being. The relationship is likely reciprocal: reduced physical capacity and emotional distress limit engagement, while impaired sexual functioning may strain relationships and diminish perceived normalcy [35,36].

Taken together, these findings support a biopsychosocial model of sexual dysfunction in MS. Neurological damage provides the substrate, fatigue functions as an important clinical amplifier, psychological distress modulates response patterns, and self-concept influences relational engagement. Within this framework, fatigue may represent a clinically meaningful component of multidimensional assessment rather than an isolated determinant of sexual dysfunction [37,38].

Several limitations should be considered. The cross-sectional design precludes causal inference and does not allow determination of temporal relationships between fatigue, psychological factors, and sexual dysfunction. The single-center setting may limit generalizability, and cultural context may influence reporting of sexual concerns. Furthermore, the study population predominantly consisted of individuals with relatively mild-to-moderate neurological disability (median EDSS = 2.0), which may limit the generalizability of the findings to patients with more advanced disease stages.

Reliance on self-report measures introduces potential reporting bias, and objective physiological assessments of sexual function were not included. The use of ASEX for categorical classification may oversimplify a dimensional phenomenon, although disease-specific MSISQ-19 assessment partially mitigates this limitation.

Residual confounding cannot be excluded, as factors such as disease-modifying therapy, hormonal status, partner variables, sleep disturbance, and pain were not incorporated into final models. In addition, medication effects—particularly antidepressants and other psychotropic treatments—may influence sexual functioning and could not be fully accounted for in the present analysis. Additionally, although hierarchical modeling enabled evaluation of incremental effects, unexplained variance indicates that sexual functioning in MS is influenced by broader neurobiological and relational mechanisms. Future longitudinal studies incorporating broader clinical, biological, and relational variables may provide a more comprehensive understanding of these interactions.

Finally, exclusion criteria enhanced internal validity but may limit representation of individuals with more advanced disease burden.

## 5. Conclusions

In conclusion, sexual dysfunction is highly prevalent in MS and reflects a multidimensional burden extending beyond neurological impairment. In the present analyses, fatigue demonstrated the most consistent associations across sexual dysfunction domains, with anxiety contributing to selected aspects of functioning. However, given the cross-sectional design, these findings should be interpreted as associations rather than causal relationships.

These findings support routine, multidimensional assessment of sexual health in MS and suggest that fatigue may represent an important component of the overall symptom burden influencing sexual functioning. Clinical approaches integrating neurological, psychological, and functional evaluation may therefore be beneficial within comprehensive MS care frameworks.

## Figures and Tables

**Table 1:** Sociodemographic and Clinical Characteristics of the Study Population.

Variable	MS (*n* = 140)	Controls (*n* = 140)	*p*-Value
**Age, years (mean ± SD)**	35.9 ± 6.0	35.6 ± 5.4	0.64
**Female sex, *n* (%)**	90 (64.3)	90 (64.3)	1
**Education level, *n* (%)**			0.412
Primary school	54 (38.6)	22 (15.7)	
High school	52 (37.1)	74 (52.9)	
University	34 (24.3)	44 (31.4)	
**Disease duration, years**	7 (0.5–15)	—	—
**Relapse number**	3 (1–20)	—	—
**EDSS score**	2.0 (1.0–6.5)	—	—

Notes: Continuous variables are presented as mean ± SD for normally distributed data and median (min–max) for non-normally distributed data. Education level comparisons were performed using the chi-square test. MS, multiple sclerosis; EDSS, Expanded Disability Status Scale; SD, standard deviation.

**Table 2:** Comparison of Sexual and Psychological Measures Between MS Patients and Controls.

Variable	MS (*n* = 140)	Controls (*n* = 140)	*p*-Value	OR (95% CI)
**Categorical variables**				
Sexual dysfunction, *n* (%)	99 (70.7)	19 (13.6)	<0.001	15.3 (8.5–27.4)
Fatigue ≥ 4, *n* (%)	93 (66.4)	7 (5.0)	<0.001	37.6 (15.8–89.4)
**Continuous variables**				
ASEX score	15 (5–28)	5 (5–21)	<0.001	—
Beck Anxiety Inventory	10 (2–35)	4 (0–18)	<0.001	—
Beck Depression Inventory	16 (3–32)	4 (0–19)	<0.001	—
Fatigue Severity Scale	4.9 (1.2–7.7)	1.6 (0.9–4.4)	<0.001	—
Body Image Scale	108 (48–182)	184 (76–200)	<0.001	—
Rosenberg Self-Esteem	30 (12–40)	35 (24–40)	<0.001	—
MSQOL-54 Physical	58.9 (22.8–92.2)	96.9 (46.1–99.5)	<0.001	—
MSQOL-54 Mental	56.3 (17.7–90.1)	93.0 (29.2–97.6)	<0.001	—

Notes: Values are presented as median (min–max) unless otherwise indicated. Continuous variables were compared using the Mann–Whitney U test and categorical variables using the chi-square test. ASEX, Arizona Sexual Experience Scale; MSQOL-54, Multiple Sclerosis Quality of Life-54; OR, odds ratio; CI, confidence interval.

**Table 3:** Comparison Within the MS Group According to Sexual Dysfunction Status.

Variable	SD Present (*n* = 99)	SD Absent (*n* = 41)	*p*-Value
**Age, years (mean ± SD)**	38.3 ± 7.9	35.0 ± 6.4	0.01
**EDSS score**	2.0 (1.0–6.5)	2.0 (2.0–2.5)	0.002
**Beck Anxiety Inventory**	12 (4–35)	7 (2–28)	<0.001
**Beck Depression Inventory**	17 (3–32)	6 (3–24)	<0.001
**Fatigue Severity Scale**	5.2 (2.0–7.7)	3.2 (1.2–5.8)	<0.001
**Body Image Scale**	88 (48–154)	140 (63–182)	<0.001
**Rosenberg Self-Esteem**	26 (14–40)	36 (12–40)	<0.001
**MSQOL-54 Physical**	53.1 (22.8–85.2)	78.1 (45.5–92.2)	<0.001
**MSQOL-54 Mental**	44.7 (17.7–83.1)	77.1 (40.6–90.1)	<0.001

Notes: Continuous variables are presented as mean ± SD for normally distributed data and median (min–max) for non-normally distributed data. Comparisons were conducted using independent samples *t*-tests or Mann–Whitney U tests as appropriate. EDSS, Expanded Disability Status Scale; MSQOL-54, Multiple Sclerosis Quality of Life-54; SD, sexual dysfunction.

**Table 4:** Hierarchical Regression Models Predicting MSISQ-19 Scores.

Outcome	Final R^2^	ΔR^2^ Attributable to Fatigue	Strongest Predictor	β	95% CI	*p*-Value
MSISQ-19 Primary	0.45	0.19	Fatigue (FSS)	0.42	0.30–0.54	<0.001
MSISQ-19 Secondary	0.336	0.13	Fatigue (FSS)	0.32	0.19–0.45	<0.001
MSISQ-19 Tertiary	0.334	0.11	Fatigue (FSS)	0.29	0.12–0.46	0.001
MSISQ-19 Total	0.453	0.18	Fatigue (FSS)	0.37	0.25–0.49	<0.001

Adjusted covariates: age, sex, education level, relapse number, EDSS score, anxiety, depression, body image, and self-esteem. Abbreviations: MSISQ-19, Multiple Sclerosis Intimacy and Sexuality Questionnaire; β, standardized regression coefficient; R^2^, coefficient of determination; ΔR^2^, change in R^2^; FSS, Fatigue Severity Scale; CI, confidence interval.

**Table 5:** Spearman Correlations Between MSISQ-19 Subscales, Psychological Measures, EDSS, and Quality of Life in MS.

Variable	1	2	3	4	5	6	7	8	9	10
**1. MSISQ-19 Primary**	1									
**2. MSISQ-19 Secondary**	0.642 **	1								
**3. MSISQ-19 Tertiary**	0.624 **	0.581 **	1							
**4. Beck Anxiety**	0.369 **	0.322 **	0.362 **	1						
**5. Beck Depression**	0.361 **	0.320 **	0.293 **	0.326 **	1					
**6. Body Image Scale**	−0.458 **	−0.425 **	−0.398 **	−0.340 **	−0.576 **	1				
**7. Rosenberg Self-Esteem**	−0.332 **	−0.302 **	−0.360 **	−0.298 **	−0.523 **	0.683 **	1			
**8. EDSS Score**	0.298 **	0.142	0.119	0.193 *	0.064	−0.147	−0.124	1		
**9. MSQOL-54 Physical**	−0.634 **	−0.539 **	−0.620 **	−0.450 **	−0.481 **	0.610 **	0.460 **	−0.226 **	1	
**10. MSQOL-54 Mental**	−0.593 **	−0.540 **	−0.602 **	−0.349 **	−0.460 **	0.564 **	0.422 **	−0.068	0.892 **	1

Notes: Values represent Spearman rho coefficients. ** *p* < 0.01, * *p* < 0.05. MSISQ-19, Multiple Sclerosis Intimacy and Sexuality Questionnaire; EDSS, Expanded Disability Status Scale; MSQOL-54, Multiple Sclerosis Quality of Life-54.

## Data Availability

The datasets generated and/or analyzed during the current study are not publicly available due to privacy and ethical restrictions but are available from the corresponding author on reasonable request.

## Supplementary Materials

The following supporting information can be downloaded at: https://www.mdpi.com/article/10.3390/jcm15062304/s1, Table S1. MSISQ-19 domain and total scores in patients with multiple sclerosis; Table S2. Fully adjusted hierarchical regression coefficients predicting MSISQ-19 domains in multiple sclerosis.

## Abbreviations

ASEX	Arizona Sexual Experience Scale
BAI	Beck Anxiety Inventory
BDI	Beck Depression Inventory
CI	Confidence Interval
EDSS	Expanded Disability Status Scale
FSS	Fatigue Severity Scale
MS	Multiple Sclerosis
MSISQ-19	Multiple Sclerosis Intimacy and Sexuality Questionnaire-19
MSQOL-54	Multiple Sclerosis Quality of Life-54
OR	Odds Ratio
SD	Sexual Dysfunction
SPSS	Statistical Package for the Social Sciences
VIF	Variance Inflation Factor

## References

[1] Boutitah-Benyaich I., Eixarch H., Villacieros-Álvarez J., Hervera A., Cobo-Calvo Á., Montalban X., Espejo C. (2025). Multiple sclerosis: Molecular pathogenesis and therapeutic intervention. Signal Transduct. Target. Ther..

[2] Alluqmani M. (2025). Multiple Sclerosis: A Comprehensive Spectrum of Symptoms Beyond Motor Dysfunction. Clin. Transl. Neurosci..

[3] Shi M., Liu Y., Gong Q., Xu X. (2024). Multiple sclerosis: An overview of epidemiology, risk factors, and serological biomarkers. Acta Neurol. Scand..

[4] Rajabi M., Shafaeibajestan S., Asadpour S., Alyari G., Taei N., Kohkalani M., Raoufinia R., Afarande H., Saburi E. (2025). Primary progressive multiple sclerosis: New therapeutic approaches. Neuropsychopharmacol. Rep..

[5] Rosso R., Virgilio E., Bronzini M., Rolla S., Maglione A., Clerico M. (2026). The Hidden Players in Multiple Sclerosis Nutrition: A Narrative Review on the Influence of Vitamins, Polyphenols, Salt, and Essential Metals on Disease and Gut Microbiota. Nutrients.

[6] Totuk O., Türkkol M., Yadi F., Güdek H.C., Erci G.Ç., Doğan İ.G., Tezer D.Ç., Sahin S., Demir S. (2025). Sexual dysfunction in multiple sclerosis: Prevalence, risk factors, and impact on quality of life in a large cohort study. BMC Neurol..

[7] Yazdani A., Ebrahimi N., Mirmosayyeb O., Ghajarzadeh M. (2023). Prevalence and risk of developing sexual dysfunction in women with multiple sclerosis (MS): A systematic review and meta-analysis. BMC Women’s Health.

[8] Brown T.A., Naragon-Gainey K. (2013). Evaluation of the unique and specific contributions of dimensions of the triple vulnerability model to the prediction of DSM-IV anxiety and mood disorder constructs. Behav. Ther..

[9] Cameron M.H., Bethoux F., Field-Fote E., Lenderking W.R., Zaiser E., Cutts K.N., Wagner J.M., Berwaerts J., Steinerman J.R. (2024). Development of an integrated conceptual model of multiple sclerosis spasticity. Disabil. Rehabil..

[10] Bol Y., Duits A.A., Lousberg R., Hupperts R.M., Lacroix M.H., Verhey F.R., Vlaeyen J.W. (2010). Fatigue and physical disability in patients with multiple sclerosis: A structural equation modeling approach. J. Behav. Med..

[11] Yeni K., Terzi M. (2025). Multiple Sclerosis-related Fatigue: An Updated Review of Pathophysiology and Associated Variables Contributing Factors. J. Mult. Scler. Res..

[12] Steward G., Looi V., Chib V.S. (2025). The Neurobiology of Cognitive Fatigue and Its Influence on Effort-Based Choice. J. Neurosci..

[13] Toccaceli Blasi M., Alfano A.R., Salzillo M., Buscarnera S., Raparelli V., Cesari M., Bruno G., Canevelli M., Initiative A.s.D.N. (2025). Sex-specific clinical and neurobiological correlates of fatigue in older adults. GeroScience.

[14] Breheny E., O’Keeffe F., Cooney S. (2026). Body image, sexual dysfunction and psychological distress in people with multiple sclerosis. Disabil. Rehabil..

[15] Stevens S.D., Thompson N.R., Sullivan A.B. (2019). Prevalence and Correlates of Body Image Dissatisfaction in Patients with Multiple Sclerosis. Int. J. MS Care.

[16] Poli S., Donisi V., das Nair R., Mazzi M.A., Gajofatto A., Rimondini M. (2025). Psychosocial Correlates of Fatigue in Young Adults with Multiple Sclerosis: Exploring the Roles of Resilience, Mindfulness, and Illness Perception. Healthcare.

[17] Scandurra C., Rosa L., Carotenuto A., Moccia M., Arena S., Ianniello A., Nozzolillo A., Turrini M., Streito L.M., Abbadessa G. (2023). Sexual dysfunction in people with multiple sclerosis: The role of disease severity, illness perception, and depression. J. Clin. Med..

[18] Mozhdehipanah H., Sharifi N., Emami A., Abbaslou M.A., Mirzadeh M. (2025). The prevalence of sexual dysfunction and its contributors among the women with multiple sclerosis. BMC Women’s Health.

[19] Soykan A. (2004). The reliability and validity of Arizona sexual experiences scale in Turkish ESRD patients undergoing hemodialysis. Int. J. Impot. Res..

[20] Mohammadi K., Rahnama P., Montazeri A., Foley F.W. (2014). The multiple sclerosis intimacy and sexuality questionnaire-19: Reliability, validity, and factor structure of the Persian version. J. Sex. Med..

[21] Idiman E., Uzunel F., Ozakbas S., Yozbatiran N., Oguz M., Callioglu B., Gokce N., Bahar Z. (2006). Cross-cultural adaptation and validation of multiple sclerosis quality of life questionnaire (MSQOL-54) in a Turkish multiple sclerosis sample. J. Neurol. Sci..

[22] Gencay-Can A., Can S.S. (2012). Validation of the Turkish version of the fatigue severity scale in patients with fibromyalgia. Rheumatol. Int..

[23] Kurnaz M.F., Koçtürk N. (2025). Adaptation of the short-form of state self-esteem scale into Turkish: A study of validity and reliability. Ank. Univ. J. Fac. Educ. Sci. (JFES).

[24] Ozcelik H.S., Ozdel K., Bulut S.D., Orsel S. (2015). The reliability and validity of the Turkish version of the Beck Scale for Suicide Ideation (Turkish BSSI). Klin. Psikofarmakol. Bülteni-Bull. Clin. Psychopharmacol..

[25] Petracca M., Carotenuto A., Scandurra C., Moccia M., Rosa L., Arena S., Ianniello A., Nozzolillo A., Turrini M., Streito L.M. (2023). Sexual dysfunction in multiple sclerosis: The impact of different MSISQ-19 cut-offs on prevalence and associated risk factors. Mult. Scler. Relat. Disord..

[26] Córdoba-Peláez M.M., Molina-Torres G., Rutkowska A., Rutkowski S., Rubio-Arias J.Á. (2025). Analysis of the Structural Characteristics and Psychometric Properties of the Multiple Sclerosis Intimacy and Sexuality Questionnaire (MSISQ-15): A Systematic Review and Meta-Analysis. Diagnostics.

[27] Romano D., Zemon V., Foley F.W. (2023). Age-related differences in the severity of sexual dysfunction symptoms and psychological distress in individuals with multiple sclerosis. Mult. Scler. Relat. Disord..

[28] Behrens M., Gube M., Chaabene H., Prieske O., Zenon A., Broscheid K.C., Schega L., Husmann F., Weippert M. (2023). Fatigue and Human Performance: An Updated Framework. Sports Med..

[29] Lane A.M., Micklewright D., Meijen C. (2025). Understanding fatigue: A psychological framework for health and performance. Sci.

[30] Braley T.J., Chervin R.D. (2010). Fatigue in multiple sclerosis: Mechanisms, evaluation, and treatment. Sleep.

[31] Nelson B.D., Hodges A., Hajcak G., Shankman S.A. (2015). Anxiety sensitivity and the anticipation of predictable and unpredictable threat: Evidence from the startle response and event-related potentials. J. Anxiety Disord..

[32] Hanna M., Strober L.B. (2020). Anxiety and depression in Multiple Sclerosis (MS): Antecedents, consequences, and differential impact on well-being and quality of life. Mult. Scler. Relat. Disord..

[33] Quinn-Nilas C., Benson L., Milhausen R.R., Buchholz A.C., Goncalves M. (2016). The Relationship Between Body Image and Domains of Sexual Functioning Among Heterosexual, Emerging Adult Women. Sex. Med..

[34] McCormack D., O’Keeffe D.F., Seery C., Eccles D.F. (2025). The association between body image and psychological outcomes in multiple sclerosis. A systematic review. Mult. Scler. Relat. Disord..

[35] Pego Pérez E.R., Bermello López M.L., Gómez Fernández E., Marín Arnés M.D.R. (2025). Predicting Quality of Life in Relapsing-Remitting Multiple Sclerosis: Clinical Burden Meets Emotional Balance in Early Disease. Neurol. Int..

[36] Dourou P., Gourounti K., Lykeridou A., Gaitanou K., Petrogiannis N., Athanasiadou C.R., Sousamli A., Xanthos T., Sarantaki A. (2024). Evaluating sexual health in women with multiple sclerosis: A study on the interplay of disability and quality of life. Healthcare.

[37] Yeni K., Tulek Z., Terzi M. (2025). Sexual dysfunction in female patients with multiple sclerosis: Relationship with functional status, fatigue, depression, sleep quality, and quality of life. J. Sex. Med..

[38] Akıncioglu S., Tavsanlı N.G., Mavioglu H. (2022). Effects of sexual dysfunction, fatigue, and Depression on the quality of life of women with multiple sclerosis. J. Mult. Scler. Res..

