# Supplementary material for: Multidimensional Determinants of Sexual Dysfunction in Multiple Sclerosis: Incremental Insights from a Hierarchical Model
Source: J Clin Med. 2026 Mar 18;15(6):2304. doi: 10.3390/jcm15062304 (PMC13027168; doi:10.3390/jcm15062304)
Supplement: Supplementary file 1 [file jcm-15-02304-s001.zip › jcm-4196872-supplementary.pdf]

**Supplementary Table S1. MSISQ-19 domain and total scores in patients with multiple sclerosis**

| MSISQ-19 outcome | MS (n = 140) | Scale range | Interpretation |
|---|---|---|---|
| Primary domain score | **13 (6–24)** | 5–25 | Neurogenic sexual dysfunction severity |
| Secondary domain score | **27 (11–42)** | 9–45 | Symptom-related sexual dysfunction severity |
| Tertiary domain score | **14 (6–23)** | 5–25 | Psychosocial sexual dysfunction severity |
| Total MSISQ-19 score | **55 (25–86)** | 19–95 | Overall sexual dysfunction severity |

***Abbreviations:*** *MS, multiple sclerosis; MSISQ-19, Multiple Sclerosis Intimacy and Sexuality Questionnaire.* ***Note:*** *Values are presented as median (minimum–maximum).*

**Supplementary Table S2. Fully adjusted hierarchical regression coefficients predicting MSISQ-19 domains in MS**

| Predictor | Primary β (p) | Secondary β (p) | Tertiary β (p) | Total β (p) |
|---|---|---|---|---|
| Age | 0.12 (0.082) | 0.09 (0.140) | 0.10 (0.118) | 0.11 (0.091) |
| Sex | 0.06 (0.310) | 0.05 (0.368) | 0.04 (0.421) | 0.05 (0.352) |
| Education | −0.08 (0.176) | −0.07 (0.211) | −0.09 (0.159) | −0.08 (0.181) |
| Relapse number | 0.09 (0.142) | 0.07 (0.214) | 0.06 (0.268) | 0.08 (0.181) |
| EDSS | 0.14 (0.058) | 0.10 (0.121) | 0.09 (0.167) | 0.13 (0.072) |
| Fatigue (FSS) | 0.42 (<0.001) | 0.32 (<0.001) | 0.29 (0.001) | 0.37 (<0.001) |
| Anxiety | 0.19 (0.012) | 0.12 (0.078) | 0.17 (0.028) | 0.21 (0.008) |
| Depression | 0.11 (0.134) | 0.10 (0.158) | 0.08 (0.211) | 0.12 (0.119) |
| Body image | −0.13 (0.074) | −0.15 (0.061) | −0.14 (0.082) | −0.16 (0.058) |
| Self-esteem | −0.10 (0.141) | −0.09 (0.172) | −0.12 (0.119) | −0.11 (0.138) |

## Model fit

| Outcome | Final $R^2$ | $\Delta R^2$ fatigue |
|---|---|---|
| Primary | 0.450 | 0.19 |
| Secondary | 0.336 | 0.13 |
| Tertiary | 0.334 | 0.11 |
| Total | 0.453 | 0.18 |

***Diagnostics:*** *VIF <3 for all predictors; Durbin–Watson 1.70–2.27.* ***Abbreviations:*** *MSISQ-19, Multiple Sclerosis Intimacy and Sexuality Questionnaire; FSS, Fatigue Severity Scale; EDSS, Expanded Disability Status Scale.*
